# Economic evaluation of alternative testing regimes and settings to detect undiagnosed HIV in Australia

**DOI:** 10.1186/s12913-020-06040-5

**Published:** 2021-01-07

**Authors:** Owain D. Williams, Judith A. Dean, Anna Crothers, Charles F. Gilks, Jeff Gow

**Affiliations:** 1 School of Public Health, Faculty of Medicine, The University of Queensland, Herston, Australia; 2 School of Medicine, Griffith University, Nathan, Australia; 3 School of Commerce, University of Southern Queensland, Toowoomba, Queensland 4350 Australia; 4 School of Accounting, Economics and Finance, University of KwaZulu-Natal, Durban, South Africa

**Keywords:** HIV testing, Conventional testing, Parallel testing, Point of care testing, Self-testing, Costs

## Abstract

**Background:**

The study aimed to estimate the comparative costs per positive diagnosis of previously undetected HIV in three testing regimes: conventional; parallel and point of care (POC) testing. The regimes are analysed in six testing settings in Australia where infection is concentrated but with low prevalence.

**Methods:**

A cost model was developed to highlight the trade-offs between test and economic efficiency from a provider perspective. First, an estimate of the number of tests needed to find a true (previously undiagnosed) positive diagnosis was made. Second, estimates of the average cost per positive diagnosis in whole of population (WoP) and men who have sex with men (MSM) was made, then third, aggregated to the total cost for diagnosis of all undetected infections.

**Results:**

Parallel testing is as effective as conventional testing, but more economically efficient. POC testing provide two significant advantages over conventional testing: they screen out negatives effectively at comparatively lower cost and, with confirmatory testing of reactive results, there is no loss in efficiency. The average and total costs per detection in WoP are prohibitive, except for Home Self Testing. The diagnosis in MSM is cost effective in all settings, but especially using Home Self Testing when the individual assumes the cost of testing.

**Conclusions:**

This study illustrates the trade-offs between economic and test efficiency and their interactions with population(s) prevalence. The efficient testing regimes and settings are presently under or not funded in Australia. Home Self Testing has the potential to dramatically increase testing rates at very little cost.

**Supplementary Information:**

The online version contains supplementary material available at 10.1186/s12913-020-06040-5.

## Background

Ambitious 90–90-90 targets have been set to diagnose 90% of all people living with HIV (PLHIV), initiate antiretroviral treatment (ART) for 90% of those diagnosed with HIV infection and to achieve an undetectable viral load in 90% of those on ART [1]. To achieve the first 90, increased levels of testing needs to occur. Of course, dynamically, as incidence falls over time due to reduced rates of transmission, the cost of detecting the last 10% of prevalence will increase. The ‘last mile problem’ is generic to infectious disease elimination campaigns [2] – especially with regard to the incremental cost-effectiveness (and incrementally elevated costs) of targeted screening that necessarily turns its focus to more hard to reach populations and infrequent or non-testers. In disease elimination strategies, the incremental costs of screening and diagnosis can be expected to rise as prevalence falls, and thus the cost-effectiveness of all available means of testing becomes a compelling area of investigation by which to gear resources and public health interventions.

The HIV prevalence in Australia among adults aged 15 years and older is estimated to be 0.14% in 2018 [3]. Transmission of HIV in Australia continues to occur primarily through male-to-male sexual contact (63% of all infections) resulting in a concentrated epidemic among men who have sex with men (MSM) (prevalence of 7.9%) [3]. In 2017, estimates suggest that 2899 (11%) people living with HIV were undiagnosed and among HIV positive MSM, 9% were undiagnosed [3]. Australia, therefore, needs to explore and consider cost effective means of detecting the last 10%.

In this context, this study aims to analyse the costs of making a HIV positive diagnosis using the three main testing regimes available electively in Queensland, Australia: conventional; parallel; and point of care (POC) testing; in six common testing settings: Private General Practice (GP); Public Sexual Health Clinic (SHC); Community Peer Organisation led General Practice (Community GP); Community Organisation led Volunteer Peer Clinic; Community Organisation led Salaried Peer Clinic; and Private at Home. The study did not consider testing in hospital settings or screening (blood and organ safety).

## Methods

### Aim

A model was developed to compare costs from the service provider perspective and to illustrate the trade-offs between test effectiveness (mainly arising from the sensitivity of the test technologies) and the economic efficiency associated with the application of these different regimes in six different testing settings.

The model seeks to demonstrate how configurations of testing technologies, regimes and settings and patient pathways can be displayed in a manner that is more informative than those generated by decision tree models. A further objective is also to generate a relatively simple model (or framework) that accommodates the sensitivities of the testing technologies used in each of three testing regimes and six settings in combination with the rates of prevalence and incidence in the whole of population (WoP) and the priority population of men who have sex with men (MSM).

### HIV testing regimes

There are three regimes that are most commonly used for elective HIV testing/screening in Australia: Conventional EIA (enzyme-linked immunoassay) laboratory testing; Parallel testing; and POC testing including HIV self-testing (HIVST). The diagnostic standard in Australia requires the reactive/positive result of the initial screening test to be confirmed by a laboratory based confirmatory Western Blot (WB) or additional supplementary blood test. Establishing a true positive diagnosis involves individuals entering one of three HIV testing regimes. Each involves various different combinations of initial and confirmatory testing technologies as part of the pathway to confirmed diagnosis.

The conventional regime is mainly used in GP clinics where the vast majority of testing for HIV and other sexually transmissible infections (STI) now occurs and in SHC [4].

In parallel testing both conventional EIA testing and rapid POC testing (DHC) are performed simultaneously at the point of testing by a health professional or appropriately trained testing facilitator [5, 6]. Parallel testing can increase the number of tests used in low prevalence countries compared to conventional testing. However, it is efficient as it enables results to be concordantly confirmed by two different initial assays at one point of service [7], thus reducing the need to wait for results or for return visits, both identified as barriers to HIV testing [8–11].

**Fig. 1:**
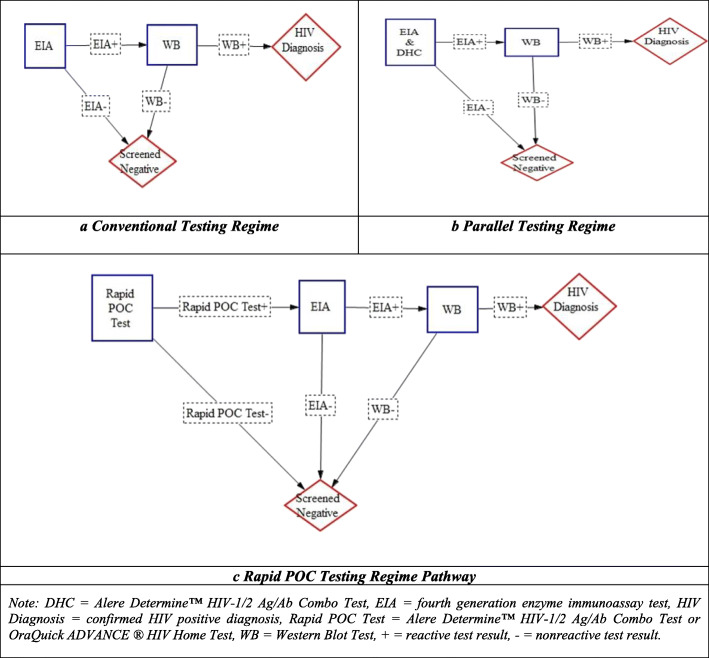
HIV Testing Regime Pathways to Confirmed Diagnosis. Note: DHC = Alere Determine™ HIV-1/2 Ag/Ab Combo Test, EIA = fourth generation enzyme immunoassay test, HIV Diagnosis = confirmed HIV positive diagnosis, Rapid POC Test = Alere Determine™ HIV-1/2 Ag/Ab Combo Test or OraQuick ADVANCE ® HIV Home Test, WB = Western Blot Test, + = reactive test result, - = nonreactive test result

The POC testing regime allows testing to occur in non-clinical settings by medical and non-medical personnel with results available at the time and place of testing or at home as is the case with HIVST [5, 12]. The rapid POC tests do not provide confirmatory diagnostic evidence of a HIV infection; and patients with reactive results require confirmation of a positive result by entering into the conventional or parallel testing regimes. The three regimes are represented diagrammatically in Fig. 1.

### HIV testing settings

All six settings were based in Brisbane, the capital city in the Australian state of Queensland, which are representative of the whole of Australia. Conventional testing pathway is conducted in SHC and Private GP setting. Parallel testing pathway is mainly available at community GP clinics, community volunteer peer clinics and community salaried peer clinics. POC testing pathway included both rapid POC testings and HIVST.

### Cost model

A cost model was constructed in Excel to compare the cost per positive diagnosis associated with two populations, three regimes and six settings and incorporating a number of inputs. Each testing regime can result in a number of possible outcomes.

For example, conventional testing could produce four possible outcomes: a true positive, a true negative, a false positive, or a false negative. Identifying all possible outcomes enables the calculation of the total costs of each outcome, the likelihood of each of these outcomes and the calculation of the average cost per person tested.

The probability of each outcome is dependent on three main model inputs: first, the probability of finding an undiagnosed HIV infection in both whole of population (WoP) and MSM; second, the sensitivity (Sen) and specificity (Spec) of each testing technology; and third, the cost inputs for each testing regime.

For example, the average cost per person tested for the conventional testing regime is calculated as:

$$ =\left({P}_{HIV}\ast Se{n}_{EIA}\ast Se{n}_{WB}\right)\ast AC\  per\  True\  HIV\  Positiv{e}_{EIA+, WB+}+\left({P}_{HIV}\ast \left(1- Se{n}_{EIA}\right)\right)\ast AC\  per\  False\  HIV\  Negativ{e}_{EIA-}+\left(\left(1-{P}_{HIV}\right)\ast Spe{c}_{EIA}\right)\ast AC\  per\  True\  HIV\  negativ{e}_{EIA-}+\left(\left(1-{P}_{HIV}\right)\ast \left(1- Spe{c}_{EIA}\right)\ast Spe{c}_{WB}\right)\ast AC\  per\  False\  HIV\  Positiv{e}_{EIA+, WB-} $$

Then the average number of tests needed to find a positive and previously undiagnosed HIV case for the conventional testing is: $=\frac{1}{P_{HIV}\ast Se{n}_{EIA}\ast Se{n}_{WB.}}$

The average cost per positive diagnosis is derived as:

$$ =\mathrm{Average}\ \mathrm{Cost}\ \mathrm{per}\ \mathrm{per}\mathrm{son}\ \mathrm{tested}\ast \mathrm{Average}\ \mathrm{number}\ \mathrm{of}\ \mathrm{tests}\ \mathrm{required}\ \mathrm{for}\ \mathrm{a}\ \mathrm{positive}\ \mathrm{diagnosis} $$

Details and assumptions for the different screening strategies modelled are presented below. The test efficiency forms part of the calculation of the probability of a finding a true undiagnosed case in each of the two populations. This factor provides a benchmark to evaluate and compare the economic efficiency of each of the three regimes and six settings.

$$ Rate\ of\ undiagnosed\  HIV\  cases\ in\ populatio{n}_i=\frac{1}{P_{HIV}} $$

### Data

#### Clinical test effectiveness

The following testing technologies and documented sensitivities and specificities were used for calculations: Architect HIV Ag/Ab Combo assay which is generally used in Australia for conventional testing [Sensitivity 99.94% (95% CI, 99.79, 100%); Specificity 99.50% (95% CI, 99.31, 99.64)] [13]; Rapid Alere Determine™ Combo widely used in Queensland for POC testing [Sensitivity 99.4% (95% CI, 96.6, 100%); Specificity 99.2% (95% CI, 98.2, 99.7)] [14]; and the OraQuick ADVANCE® HIV Home Test for self-testing as this particular kit is the closest to market approval in Australia [Sensitivity 91.67% (95% CI, 84.24, 96.33%); Specificity 99.98% (95% CI, 99.89, 100)] [15].

#### Probability of detecting an undiagnosed HIV infection

The probability of detection is derived by deduction from the estimates of the number of undiagnosed HIV positive cases.

$$ \mathrm{Probability}\ \mathrm{of}\ \mathrm{finding}\ \mathrm{an}\ \mathrm{undiagnosed}\ \mathrm{HIV}\ \mathrm{case}\ \mathrm{in}\ \mathrm{populatio}{\mathrm{n}}_{\mathrm{i}}=\frac{\mathrm{Number}\ \mathrm{of}\ \mathrm{undiagnosed}\ \mathrm{HIV}\ \mathrm{case}\mathrm{s}\ \mathrm{in}\ {\mathrm{population}}_{\mathrm{i}}}{{\mathrm{population}}_{\mathrm{i}}\ \mathrm{s}\mathrm{ize}} $$

In 2014, the Australian WoP aged 15 years or greater was 18,754,954 [16]. The MSM population was estimated at 305,783 using data from the 2nd Australian Study of Health and Relationships Survey [17] and demographic information from the Australian Bureau of Statistics [16]. It should be noted that there are limited data available on the relative size and distribution of the MSM population in the states and territories of Australia, and any enumeration of this population at the sub-national level is complex and subject to certain assumptions. Thus, there is a high degree of uncertainty in these estimates.

Data on HIV incidence and estimated undiagnosed prevalence for both the WoP and MSM populations was derived from the Australia Annual HIV Surveillance Report published in 2015 [18]. National rates of undiagnosed HIV prevalence were used because only national population estimates for undiagnosed prevalence in the general and MSM population are available. Additionally, the latest survey data reports only national prevalence of the number of men who identify as MSM. The number of undiagnosed HIV positive people in the general population in 2014 was estimated to be 3350 (CI 95%, 2100-4670) and in the MSM population 1848 (CI 95%, 850–2896). Thus, mass universal testing in WoP would yield a 0.0179% probability of finding an undiagnosed HIV infection per person tested and a 0.6044% probability in the MSM population assuming everyone has an equal chance of being tested.

#### Cost data collection

From February to April 2016 costing information was collected from community sites, SHC and GP clinics. Costs incurred by the health system include: consult length; staff salary; consult costs including Medicare Benefits Schedule rebates from the publicly funded universal health care system operated by the Commonwealth of Australia Department of Human Services; test costs; and confirmatory laboratory testing for reactive results. Costs were calculated from the health care provider’s perspective, with the exception of HIVST, which was based on the retail price of the test kit established elsewhere [19].

The cost of testing in the six settings and by HIV status are outlined in Tables 1, 2, 3, 4, 5 and 6.

**Table 1:** Conventional Testing Regime: Private General Practice Clinic Setting – (Funded by Federal Government – Medicare)

	Probability of True HIV Positive (***P***_***HIV***_)		Probability of True HIV Negative (1 ***− P***_***HIV***_)	
Outcomes	HIV Positive (EIA+ & WB+)	False HIV Negative (EIA-)	False HIV Positive (EIA+ & WB-)	True HIV Negative (EIA-)
Probability of result	(*P*_*HIV*_ ∗ *Sen*_*EIA*_ ∗ *Sen*_*WB*_)	*P*_*HIV*_ ∗ (1 − *Sen*_*EIA*_)	((1 − *P*_*HIV*_) ∗ (1 − *Spec*_*EIA*_) ∗ *Spec*_*WB*)_	(1 − *P*_*HIV*_) ∗ *Spec*_*EIA*_
**Inputs**				
**Visit 1: Initial Screening**				
Consult time	20 min	20 min	20 min	20 min
Medicate Rebate	$71.70^1^	$71.70^1^	$71.70^1^	$71.70^1^
Staffing	GP	GP	GP	GP
**Pathology costs**				
EIA	$15.65^2^	$15.65^2^	$15.65^2^	$15.65^2^
WB confirmatory	$29.00^3^		$29.00^3^	
**Visit 2: Test Results**				
Consult time	40 min	20 min	20 min	20 min
Medicate Rebate	$105.55^4^	$71.70^1^	$71.70^1^	$71.70^1^
Staffing	GP	GP	GP	GP
**Total Cost of Each Outcome**	$221.90	$159.05	$188.05	$159.05
**Average cost (AC) per person tested**	=(*P*_*HIV*_ ∗ *Sen*_*EIA*_ ∗ *Sen*_*WB*_) ∗ *AC per True HIV Positive*_*EIA* + , *WB*+_ + (*P*_*HIV*_ ∗ (1 − *Sen*_*EIA*_)) ∗ *AC per False HIV Negative*_*EIA*−_ + ((1 − *P*_*HIV*_) ∗ *Spec*_*EIA*_) ∗ *AC per True HIV Negative*_*EIA*−_ + ((1 − *P*_*HIV*_) ∗ (1 − *Spec*_*EIA*_) ∗ *Spec*_*WB*_)) ∗ *AC per False HIV Positive*_*EIA* + , *WB*−_			
**Average number of tests for a positive diagnosis**	$=\frac{1}{P_{HIV}\ast Se{n}_{EIA}\ast Se{n}_{WB}}$			

Note: *AC* Average Cost, *EIA* fourth generation enzyme immunoassay test, *GP* General Practitioner, *P* Probability, *Sen* Sensitivity, *Spec* Specificity, *WB* Western Blot Test, + reactive test result, − nonreactive test result

1 = Medicare Rebate Item 36; 2 = Medicare Rebate Item 69,384; 3 = Medicare Rebate Item 69,387; 4 = Medicare Rebate Item 44

**Table 2:** Conventional Testing Regime: Public Sexual Health Clinic Setting (Funded by State Government)

	True HIV Positive (***P***_***HIV***_ )		True HIV Negative (1 ***− P***_***HIV***_)	
Outcomes	HIV Positive (EIA+ & WB+)	False HIV Negative (EIA-)	False HIV Positive (EIA+ & WB-)	True HIV Negative (EIA-)
Probability of result	(*P*_*HIV*_ ∗ *Sen*_*EIA*_ ∗ *Sen*_*WB* )_	(*P*_*HIV*_ ∗ (1 − *Sen*_*EIA*_))	((1 − *P*_*HIV*_) ∗ (1 − *Spec*_*EIA*_) ∗ *Spec*_*WB*_)	(1 − *P*_*HIV*_) ∗ *Spec*_*EIA*_
**Inputs**				
**Visit 1: Initial Screening**				
Consult time	30 Min	30 Min	30 Min	30 Min
Staff Wage (Rate/Hour):	$45.12	$45.12	$45.12	$45.12
Staffing	Clinical Nurse	Clinical Nurse	Clinical Nurse	Clinical Nurse
**Pathology costs**				
EIA	$18.54	$18.54	$18.54	$18.54
WB confirmatory	$84.54		$84.54	
**Visit 2: Test Results**				
Consult time	60 Min	30 Min	30 Min	30 Min
Staff Wage (Rate/Hour):	$78.00	$45.12	$45.12	$45.12
Staffing	Staff specialist (Level 18)	Clinical Nurse	Clinical Nurse	Clinical Nurse
**Total Cost of Each Outcome**	$203.64	$63.67	$148.21	$63.67
**Average cost per person tested**	=(*P*_*HIV*_ ∗ *Sen*_*EIA*_ ∗ *Sen*_*WB*_) ∗ *AC per True HIV Positive*_*EIA* + , *WB*+_ + (*P*_*HIV*_ ∗ (1 − *Sen*_*EIA*_)) ∗ *AC per False HIV Negative*_*EIA*−_ + ((1 − *P*_*HIV*_) ∗ *Spec*_*EIA*_) ∗ *AC per True HIV Negative*_*EIA*−_ + ((1 − *P*_*HIV*_) ∗ (1 − *Spec*_*EIA*_) ∗ *Spec*_*WB*_)) ∗ *AC per False HIV Positive*_*EIA* + , *WB*−_			
**Average number of tests for a positive diagnosis**	$=\frac{1}{P_{HIV}\ast Se{n}_{EIA}\ast Se{n}_{WB}}$			

Note: *AC* Average Cost, *EIA* fourth generation enzyme immunoassay test, *P* Probability, *Sen* Sensitivity, *Spec* Specificity, *WB* Western Blot Test, + reactive test result, − nonreactive test result

**Table 3:** Parallel Testing Regime: Community Peer Organisation Bulk Billing GP Clinic Setting (Funded by Federal Government - Medicare and State Government)

	True HIV Positive (***P***_***HIV***_)				True HIV Negative (1 ***− P***_***HIV***_)			
Outcomes	DHC+, EIA+, WB+	DHC-, EIA+, WB+	DHC+, EIA-	DHC-, EIA-	DHC+, EIA-	DHC+, EIA+, WB-	DHC-, EIA+,WB-	DHC-, EIA-
Probability of result	(*P*_*HIV*_ ∗ *Sen*_*DHC*_ ∗ *Sen*_*EIA*_ ∗ *Sen*_*WB*_)	(*P*_*HIV*_ ∗ (1 − *Sen*_*DHC*_) ∗ *Sen*_*EIA*_ ∗ *Sen*_*WB* )_	(*P*_*HIV*_ ∗ *Sen*_*DHC*_ ∗ (1 − *Sen*_*EIA*_))	(*P*_*HIV*_∗ (1 − *Sen*_*DHC*_) ∗ (1 − *Sen*_*EIA*_))	((1 − *P*_*HIV*_) ∗ (1 − *Spec*_*DHC*_) ∗ *Spec*_*EIA*_)	((1 − *P*_*HIV*_) ∗ (1 − *Spec*_*DHC*_) ∗ (1 − *Spec*_*EIA*_) ∗ *Spec*_*WB* )_	((1 − *P*_*HIV*_) ∗ *Spec*_*DHC*_ ∗ (1 − *Spec*_*EIA*_) ∗ *Spec*_*WB* )_	((1 − *P*_*HIV*_) ∗ *Spec*_*DHC*_ ∗ *Spec*_*EIA* )_
**Inputs**								
**Visit 1: Initial Screening**								
Consult time	20 min	20 min	20 min	20 min	20 min	20 min	20 min	20 min
Medicate Rebate	$71.70^1^	$71.70^1^	$71.70^1^	$71.70^1^	$71.70^1^	$71.70^1^	$71.70^1^	$71.70^1^
Staffing	GP	GP	GP	GP	GP	GP	GP	GP
**Pathology costs**								
Rapid Alere Determine Test (DHC)	$11	$11	$11	$11	$11	$11	$11	$11
EIA	$15.65^2^	$15.65^2^	$15.65^2^	$15.65^2^	$15.65^2^	$15.65^2^	$15.65^2^	$15.65^2^
WB confirmatory	$29.00^3^	$29.00^3^				$29.00^3^	$29.00^3^	
**Visit 2: Test Results**								
Consult time	40 min	40 min	20 min		20 min	20 min	20 min	
Medicare Rebate	$105.55^4^	$105.55^4^	$71.70^1^		$71.70^1^	$71.70^1^	$71.70^1^	
Staffing	GP	GP	GP		GP	GP	GP	
**Total Cost of Each Outcome**	$232.90	$232.90	$170.05	$98.35	$170.05	$199.05	$199.05	$98.35
**Average cost per person tested**	=(*P*_*HIV*_ ∗ *Sen*_*DHC*_ ∗ *Sen*_*EIA*_ ∗ *Sen*_*WB*_) ∗ *AC per True HIV Positive*_*DHC* + , *EIA* + , *WB*+_ + (*P*_*HIV*_ ∗ (1 − *Sen*_*DHC*_) ∗ *Sen*_*EIA*_ ∗ *Sen*_*WB*_) ∗ *AC per True HIV Positive*_*DHC* − , *EIA* + , *WB*+_ + (*P*_*HIV*_ ∗ *Sen*_*DHC*_ ∗ (1 − *Sen*_*EIA*_)) ∗ *AC per False Negative*_*DHC* + , *EIA*−_ + (*P*_*HIV*_ ∗ (1 − *Sen*_*DHC*_) ∗ (1 − *Sen*_*EIA*_)) ∗ *AC per False Negative*_*DHC* − , *EIA*−_ + ((1 − *P*_*HIV*_) ∗ (1 − *Spec*_*DHC*_) ∗ *Spec*_*EIA*_) ∗ *AC per False HIV Positive*_*DHC* + , *EIA*−_ + ((1 − *P*_*HIV*_) ∗ (1 − *Spec*_*DHC*_) ∗ (1 − *Spec*_*EIA*_) ∗ *Spec*_*WB*_) ∗ *AC per False HIV Positive*_*DHC* + , *EIA* + , *WB*−_ + ((1 − *P*_*HIV*_) ∗ *Spec*_*DHC*_ ∗ (1 − *Spec*_*EIA*_) ∗ *Spec*_*WB*_) ∗ *AC per False HIV Positive*_*DHC* − , *EIA* + , *WB*−_ + ((1 − *P*_*HIV*_) ∗ *Spec*_*DHC*_ ∗ *Spec*_*EIA*_) ∗ *AC per True Negative*_*DHC* − , *EIA*−_							
**Average number of tests for a positive diagnosis**	$=\frac{1}{\left({P}_{HIV}\ast Se{n}_{DHC}\ast Se{n}_{EIA}\ast Se{n}_{WB}\right)+\left({P}_{HIV}\ast \left(1- Se{n}_{DHC}\right)\ast Se{n}_{EIA}\ast Se{n}_{WB}\right)}$							

Note: *AC* Average Cost, *DHC* Alere Determine™ HIV-1/2 Ag/Ab Combo Test, *EIA* fourth generation enzyme immunoassay test, *GP* General Practitioner, *P* Probability, *Sen* Sensitivity, *Spec* Specificity, *WB* Western Blot Test, + reactive test result, − nonreactive test result. 1 = Medicare Rebate Item 36; 2 = Medicare Rebate Item 69,384; 3 = Medicare Rebate Item 69,387; 4 = Medicare Rebate Item 44

**Table 4:** Parallel Testing Regime: Community Peer Testing Service using Volunteer Peer Nurse Setting (Funded by Federal Government - Medicare and State Government)

	True HIV Positive (***P***_***HIV***_)				True HIV Negative (1 ***− P***_***HIV***_)			
Outcomes	DHC+, EIA+, WB+	DHC-, EIA+, WB+	DHC+, EIA-	DHC-, EIA-	DHC+, EIA-	DHC+, EIA+, WB-	DHC-, EIA+, WB-	DHC-, EIA-
Probability of result	(*P*_*HIV*_ ∗ *Sen*_*DHC*_ ∗ *Sen*_*EIA*_ ∗ *Sen*_*WB* )_	(*P*_*HIV*_ ∗ (1 − *Sen*_*DHC*_) ∗ *Sen*_*EIA*_ ∗ *Sen*_*WB* )_	(*P*_*HIV*_ ∗ *Sen*_*DHC*_ ∗ (1 − *Sen*_*EIA*_))	(*P*_*HIV*_∗ (1 − *Sen*_*DHC*_) ∗ (1 − *Sen*_*EIA*_))	((1 − *P*_*HIV*_) ∗ (1 − *Spec*_*DHC*_) ∗ *Spec*_*EIA*_ )	((1 − *P*_*HIV*_) ∗ (1 − *Spec*_*DHC*_) ∗ (1 − *Spec*_*EIA*_) ∗ *Spec*_*WB* )_	((1 − *P*_*HIV*_) ∗ *Spec*_*DHC*_ ∗ (1 − *Spec*_*EIA*_) ∗ *Spec*_*WB*_ )	((1 − *P*_*HIV*_) ∗ *Spec*_*DHC*_ ∗ *Spec*_*EIA* )_
**Inputs**								
**Visit 1: Initial Screening – Volunteer Peer Testing Facilitators**								
Consult time	20 min	20 min	20 min	20 min	20 min	20 min	20 min	20 min
Staff Wage (Rate/Hour):	$0	$0	$0	$0	$0	$0	$0	$0
Staffing	Volunteer Nurse	Volunteer Nurse	Volunteer Nurse	Volunteer Nurse	Volunteer Nurse	Volunteer Nurse	Volunteer Nurse	Volunteer Nurse
**Pathology costs**								
Rapid Alere Determine Test	$11	$11	$11	$11	$11	$11	$11	$11
EIA test	$15.65^1^	$15.65^1^	$15.65^1^	$15.65^1^	$15.65^1^	$15.65^1^	$15.65^1^	$15.65^1^
WB confirmatory	$29.00^2^	$29.00^2^				$29.00^2^	$29.00^2^	
**Visit 2: Test Results – Community Peer Organisation Bulk Billing GP Clinic**								
Consult time	40 min	40 min	20 min		20 min	20 min	20 min	
Medicate Rebate	$105.55^3^	$105.55^3^	$71.70^4^		$71.70^4^	$71.70^4^	$71.70^4^	
Staffing	GP	GP	GP		GP	GP	GP	
**Total Cost of Each Outcome**	$161.20	$161.20	$98.35	$26.65	$98.35	$127.35	$127.35	$26.65
**Average cost per person tested**	=(*P*_*HIV*_ ∗ *Sen*_*DHC*_ ∗ *Sen*_*EIA*_ ∗ *Sen*_*WB*_) ∗ *AC per True HIV Positive*_*DHC* + , *EIA* + , *WB*+_ + (*P*_*HIV*_ ∗ (1 − *Sen*_*DHC*_) ∗ *Sen*_*EIA*_ ∗ *Sen*_*WB*_) ∗ *AC per True HIV Positive*_*DHC* − , *EIA* + , *WB*+_ + (*P*_*HIV*_ ∗ *Sen*_*DHC*_ ∗ (1 − *Sen*_*EIA*_)) ∗ *AC per False Negative*_*DHC* + , *EIA*−_ + (*P*_*HIV*_ ∗ (1 − *Sen*_*DHC*_) ∗ (1 − *Sen*_*EIA*_)) ∗ *AC per False Negative*_*DHC* − , *EIA*−_ + ((1 − *P*_*HIV*_) ∗ (1 − *Spec*_*DHC*_) ∗ *Spec*_*EIA*_) ∗ *AC per False HIV Positive*_*DHC* + , *EIA*−_ + ((1 − *P*_*HIV*_) ∗ (1 − *Spec*_*DHC*_) ∗ (1 − *Spec*_*EIA*_) ∗ *Spec*_*WB*_) ∗ *AC per False HIV Positive*_*DHC* + , *EIA* + , *WB*−_ + ((1 − *P*_*HIV*_) ∗ *Spec*_*DHC*_ ∗ (1 − *Spec*_*EIA*_) ∗ *Spec*_*WB*_) ∗ *AC per False HIV Positive*_*DHC* − , *EIA* + , *WB*−_ + ((1 − *P*_*HIV*_) ∗ *Spec*_*DHC*_ ∗ *Spec*_*EIA*_) ∗ *AC per True Negative*_*DHC* − , *EIA*−_							
**Average number of tests for a positive diagnosis**	$=\frac{1}{\left({P}_{HIV}\ast Se{n}_{DHC}\ast Se{n}_{EIA}\ast Se{n}_{WB}\right)+\left({P}_{HIV}\ast \left(1- Se{n}_{DHC}\right)\ast Se{n}_{EIA}\ast Se{n}_{WB}\right)}$							

Note: *AC* Average Cost, *DHC* Alere Determine™ HIV-1/2 Ag/Ab Combo Test, *EIA* fourth generation enzyme immunoassay test, *GP* General Practitioner, *P* Probability, *Sen* Sensitivity, *Spec* Specificity, *WB* Western Blot Test, + reactive test result, − nonreactive test result. 1 = Medicare Rebate Item 69,384; 2 = Medicare Rebate Item 69,387; 3 = Medicare Rebate Item 44; 4 = Medicare Rebate Item 36

**Table 5:** Point of Care Testing Regime: Community Peer Testing Service Setting (Funded by State Government)

	True HIV Positive (***P***_***HIV***_)			True HIV Negative (1 ***− P***_***HIV***_)		
Outcomes	DHC+, EIA+, WB+	DHC-	DHC+, EIA-	DHC+, EIA+, WB-	DHC+ EIA-	DHC-
Probability of result	(*P*_*HIV*_ ∗ *Sen*_*DHC*_ ∗ *Sen*_*EIA*_ ∗ *Sen*_*WB* )_	(*P*_*HIV*_ ∗ (1 − *Sen*_*DHC*_))	(*P*_*HIV*_ ∗ *Sen*_*DHC*_ ∗ (1 − *Sen*_*EIA*_))	((1 − *P*_*HIV*_) ∗ (1 − *Spec*_*DHC*_) ∗ (1 − *Spec*_*EIA*_) + *Spec*_*WB*_ )	((1 − *P*_*HIV*_) ∗ (1 − *Spec*_*DHC*_) ∗ *Spec*_*EIA* )_	(1 − *P*_*HIV*_) ∗ *Spec*_*DHC*_
**Inputs**						
**Visit 1: Initial Screening - Community Peer Testing Service**						
Consult time	60 Min	30 Min	60 Min	60 Min	60 Min	30 Min
Staff Wage (Rate/Hour):	$45	$45	$45	$45	$45	$45
Staffing	Peer	Peer	Peer	Peer	Peer	Peer
**Pathology costs**						
Rapid Alere (DHC) Determine Test	$11	$11	$11	$11	$11	$11
**Referral to Bulk Billing GP for Confirmatory Testing**						
**Visit 1: Initial Screening**						
Consult time	20 min		20 min	20 min	20 min	
Medicate Rebate	$71.70^1^		$71.70^1^	$71.70^1^	$71.70^1^	
Staffing	GP		GP	GP	GP	
**Pathology costs**						
EIA test	$15.65^2^		$15.65^2^	$15.65^2^	$15.65^2^	
WB confirmatory	$29.00^3^			$29.00^3^		
**Visit 2: Test Results**						
Consult time	40 min		20 min	20 min	20 min	
Medicate Rebate	$105.55^4^		$71.70^1^	$71.70^1^	$71.70^1^	
Staffing	GP		GP	GP	GP	
**Total Cost of Each Outcome**	$277.90	$33.50	$215.05	$244.05	$215.05	$33.50
**Average cost per person tested**	=(*P*_*HIV*_ ∗ *Sen*_*DHC*_ ∗ *Sen*_*EIA*_ ∗ *Sen*_*WB*_) ∗ *AC per True Positive*_*DHC* + , *EIA* + , *WB*+_ + (*P*_*HIV*_ ∗ (1 − *Sen*_*DHC*_)) ∗ *AC per False Negative*_*DHC*−_ + (*P*_*HIV*_ ∗ *Sen*_*DHC*_ ∗ (1 − *Sen*_*EIA*_)) ∗ *AC per False Negative*_*DHC* + , *EIA*−_ + ((1 − *P*_*HIV*_) ∗ *Spec*_*DHC*_) ∗ *AC True Negative*_*DHC*−_ + ((1 − *P*_*HIV*_) ∗ (1 − *Spec*_*DHC*_) ∗ *Spec*_*EIA*_) ∗ *False Positive*_*DHC* + , *EIA*−_ + ((1 − *P*_*HIV*_) ∗ (1 − *Spec*_*DHC*_) ∗ (1 − *Spec*_*EIA*_) ∗ *Spec*_*WB*_) ∗ *False Positive*_*DHC* + , *EIA* + , *WB*−_					
**Average number of tests for a positive diagnosis**	$=\frac{1}{P_{HIV}\ast Se{n}_{DHC}\ast Se{n}_{EIA}\ast Se{n}_{WB}}$					

Note: *AC* Average Cost, *DHC* Alere Determine™ HIV-1/2 Ag/Ab Combo Test, *EIA* fourth generation enzyme immunoassay test, *GP* General Practitioner, *P* Probability, *Sen* Sensitivity, *Spec* Specificity, *WB* Western Blot Test, + = reactive test result, − = nonreactive test result. 1 = Medicare Rebate Item 36; 2 = Medicare Rebate Item 69,384; 3 = Medicare Rebate Item 69,387; 4 = Medicare Rebate Item 44

**Table 6:** Point of Care Testing Regime: Home HIVST Setting (Funded by Consumer)

	True HIV Positive (***P***_***HIV***_)			True HIV Negative (1 ***− P***_***HIV***_)		
Outcomes	HIVST+, EIA+, WB+	HIVST -	HIVST +, EIA-	HIVST+, EIA+, WB-	HIVST+ EIA-	HIVST -
Probability of result	(*P*_*HIV*_ ∗ *Sen*_*HIVST*_ ∗ *Sen*_*EIA*_ ∗ *Sen*_*WB* )_	*P*_*HIV*_ ∗ (1 − *Sen*_*HIVST*_)	(*P*_*HIV*_ ∗ *Sen*_*HIVST*_ ∗ (1 − *Sen*_*EIA*_))	((1 − *P*_*HIV*_) ∗ (1 − *Spec*_*HIVST*_) ∗ (1 − *Spec*_*EIA*_) + *Spec*_*WB*_ )	((1 − *P*_*HIV*_) ∗ (1 − *Spec*_*HIVST*_) ∗ *Spec*_*EIA* )_	(1 − *P*_*HIV*_) ∗ *Spec*_*HIVST*_
**Inputs**						
**Visit 1: Initial Screening**						
Staffing	Self	Self	Self	Self	Self	Self
OraQuick® In-Home HIV Test	$54.75	$54.75	$54.75	$54.75	$54.75	$54.75
Postage	$7.50	$7.50	$7.50	$7.50	$7.50	$7.50
**Confirmatory Testing conducted at Bulk Billing GP**						
**Visit 1: Initial Screening**						
Consult time	20 min		20 min	20 min	20 min	
Medicare Rebate	$71.70^1^		$71.70^1^	$71.70^1^	$71.70^1^	
Staffing	GP		GP	GP	GP	
**Pathology costs**						
EIA test	$15.65^2^		$15.65^2^	$15.65^2^	$15.65^2^	
WB confirmatory	$29.00^3^			$29.00^3^		
**Visit 2: Test Results**						
Consult time	40 min		20 min	20 min	20 min	
Medicare Rebate	$105.55^4^		$71.70^1^	$71.70^1^	$71.70^1^	
Staffing	GP		GP	GP	GP	
**Total Cost of Each Outcome**	$284.15	$62.25	$221.30	$250.30	$221.30	$62.25
**Total Cost of Each Outcome (excluding private costs)**	*$221.90*	*$0*	*$159.05*	*$188.05*	*$159.05*	*$0*
**Average cost per person tested**	=(*P*_*HIV*_ ∗ *Sen*_*HIVST*_ ∗ *Sen*_*EIA*_ ∗ *Sen*_*WB*_) ∗ *AC per True Positive*_*HIVST* + , *EIA* + , *WB*+_ + (*P*_*HIV*_ ∗ (1 − *Sen*_*HIVST*_)) ∗ *AC per False Negative*_*HIVST*−_ + (*P*_*HIV*_ ∗ *Sen*_*HIVST*_ ∗ (1 − *Sen*_*EIA*_)) ∗ *AC per False Negative*_*HIVST* + , *EIA*−_ + ((1 − *P*_*HIV*_) ∗ *Spec*_*HIVST*_) ∗ *AC True Negative*_*HIVST*−_ + ((1 − *P*_*HIV*_) ∗ (1 − *Spec*_*HIVST*_) ∗ *Spec*_*EIA*_) ∗ *False Positive*_*HIVST* + , *EIA*−_ + ((1 − *P*_*HIV*_) ∗ (1 − *Spec*_*HIVST*_) ∗ (1 − *Spec*_*EIA*_) ∗ *Spec*_*WB*_) ∗ *False Positive*_*HIVST* + , *EIA* + , *WB*−_					
**Average number of tests for a positive diagnosis**	$=\frac{1}{P_{HIV}\ast Se{n}_{HIV ST}\ast Se{n}_{EIA}\ast Se{n}_{WB}}$					

Note: *AC* Average Cost, *DHC* Alere Determine™ HIV-1/2 Ag/Ab Combo Test, *EIA* fourth generation enzyme immunoassay test, *GP* General Practitioner, *P* Probability, *Sen* Sensitivity, *Spec* Specificity, *WB* Western Blot Test, + reactive test result, − nonreactive test result. 1 = Medicare Rebate Item 36; 2 = Medicare Rebate Item 69,384; 3 = Medicare Rebate Item 69,387; 4 = Medicare Rebate Item 44

## Results

### Test efficiency in detecting undiagnosed HIV cases

Conventional and parallel testing regimes were found to be more efficient (higher sensitivity and specificity) in detecting a confirmed positive HIV diagnosis than POC testing, resulting in the number of tests required to detect HIV being lower for the former regimes compared to the latter (Tables A1 to A6b in Additional file 1). Conventional, parallel and Point of Care testing conducted at a community organisation each detect undiagnosed cases at an approximate rate of one per 5602 and one per 5787 per person tested respectively, and in WoP at 165 and 171, respectively in the MSM population. In comparison, home Self Testing results in slightly higher rates at one per 6111 per person in the WoP and one per 180 in the MSM population.

### Costs

Costs vary across regimes and settings depending on the number of visits, labour costs and types of labour used, and the number (if any) of pathology tests required.

The estimated cost per HIV diagnosis in the six settings for WoP were found to be extremely high, except for HIVST, when private costs were ignored (Table 7). Secondly, the cost per diagnosis for the MSM population were considerably lower due to the higher prevalence and therefore ease of detection, compared to WoP (Table 8).

**Table 7:** Costs per Undetected HIV Diagnosis in the Whole of Population (WoP)

Estimated Number of Undiagnosed Cases	3350			
Regime and Setting	Number of Tests Needed per Positive Diagnosis	Average Cost per Test Regime AUD$	Total Cost per Positive HIV Diagnosis AUD$	
**Conventional Testing Regime**				
Private General Practice	5602	$159.10	$891,329	
Public Sexual Health Clinic	5602	$63.80	$357,411	
**Parallel Testing Regime**				
Community Organisation (General Practice)	5787	$98.92	$572,542	
Community Organisation (Peer Testing - Volunteer)	5787	$27.14	$157,071	
**Point of Care Testing Regime**				
Community Organisation (Peer Testing – Paid)	5787	$34.63	$200,436	
Home HIVST (include private costs)	6111	$62.32	$380,860	
Home HIVST (exclude private costs)	6111	$0.07	$416

**Table 8:** Costs per Undetected HIV Diagnosis in the MSM Population

Estimated Number of Undiagnosed Cases	1848		
Regime and Setting	Number of Tests Needed per Positive Diagnosis	Average Cost per Test Regime AUD$	Total Cost per Positive HIV Diagnosis AUD$
**Conventional Testing Regime**			
Private General Practice	165	$159.46	$26,399
Public Sexual Health Clinic	165	$64.62	$10,697
**Parallel Testing Regime**			
Community Organisation (General Practice)	171	$99.71	$17,053
Community Organisation (Peer Testing - Volunteer)	171	$27.92	$4776
**Point of Care Testing Regime**			
Community Organisation (Peer Testing – Paid)	171	$36.01	$6159
Home HIVST (include private costs)	180	$63.51	$11,469
Home HIVST (exclude private costs)	180	$1.26	$227

The results of this study demonstrate that both test effectiveness and economic efficiency are constituents in comparing cost per HIV diagnosis in all testing settings. First, it is clear that clinicians’ wages are significant drivers of the costs of individual HIV testing and its detection. Conventional testing with clinical nurses and parallel testing with volunteer peer testers are three times more cost effective than either regime with GP/doctor’s wages. Parallel testing saves costs compared to the GP/doctor conventional setting because there are no follow-on visits for negative rapid results. There is, again, no loss of test effectiveness in the model due to the combination of conventional and POC testing being used for those with an initial reactive result.

Second, public SHC are far less costly (at less than half the cost) than conventional GP testing, largely due to the substantial salary savings of using nurses in combination with doctor/medical officers where the latter are only present for the event of a positive diagnosis.

Third, the peer rapid POC testing clinic is less expensive than almost all settings because it requires clients to only attend the clinic for an initial test overseen by a low cost peer tester, unless in the case of a reactive result when clients are referred for confirmatory testing and require a doctor/GP services. The standard rapid POC test results in a relatively small loss in test effectiveness. However, the average cost per test is significantly lower because of a combination of the lower peer salary levels compared to nurse and doctor/medical officers including GPs, and there is no need for a follow up visit to receive a result (either non-reactive or reactive) even when testing a comparatively higher prevalence MSM clientele. What primarily determines cost effectiveness of POC settings, is how inexpensive it is to screen negatives and not how expensive it is to diagnose positives.

### Sensitivity analysis

This was conducted on variables of interest: HIV prevalence levels; sensitivity and specificity of the testing technologies as well as labour time and costs. No significant (> 10%) variation was evidenced in the cost results as a result of changes to any of the parameters.

## Discussion

The cost results demonstrate that testing with rapid POC technologies, both peer facilitated and performed in the home/private, are the most cost effective means of achieving undiagnosed HIV diagnosis in WoP and MSM populations. This result holds irrespective of the losses in test effectiveness caused by the relatively lower sensitivity of the POC tests. Thus, in low prevalence countries like Australia, the biggest cost consideration for whole of population HIV screening should be how cheaply a screening modality can identify and diagnose negative cases, rather than how cheaply it can detect positive cases. Testing regimes that can screen but cannot confirm a diagnosis are highly cost effective. For example, community clinics using rapid POC testing can screen a negative case for under $35 and confirm a positive case for less than $300, whilst HIVST is dramatically less expensive, with screening of negative cases is of a negligible cost especially with government supplied ‘free’ POC test kits.

Costs per diagnosis rise dramatically when prevalence is low [20]. Screening low-risk populations with more expensive and sensitive technologies is not cost-effective when prevalence is low [21]. Thus, it would make sense to use cheaper and less efficient (or effective) test technologies if cost is the only consideration. For example, results of this study suggests a HIV diagnosis made in a GP practice costs almost $900,000, but a HIV diagnosis made in a MSM community clinic using POC costs less than $7000.

The focus on cost per diagnosis in other studies often fails to provide a breakdown of what are the constituent drivers of cost or what were the factor inputs into the particular model (in this study these were prevalence, cost inputs and test effectiveness). Most studies either compare different testing regimes in the same clinical setting [22, 23] or the same regime in different clinic settings [24, 25]. This study therefore provides an alternative costing model as it compares costs in different regimes and testing settings. The methodology applied in this study is important as HIV testing services are continually evolving to be delivered in different settings and by different people as testing technologies emerge and patterns of HIV infection, risk and population testing needs change [26]. The only comparative self-testing cost study is one which recently reported in the U.S. and has shown comparable values to those reported here [27]. The U.S. values were $US61 per self-test completed; and an incremental cost per new HIV diagnosis of $US9365 [27].

In this study a novel model has been presented by which to evaluate HIV testing regimes in a more flexible and easy manner than has been achieved previously. For example, decision tree models commonly use cost inputs or other bases of calculations which can be opaque. The model reported in this study identifies all possible outcomes and test results that a particular regime might lead to, and the cost inputs that these might incur, with population prevalence incorporated as a cost variable. This allows the cost of testing regime, settings and technologies to be established from a provider perspective. The cost incurred for a true positive diagnosis has been lowered by the adoption of less sensitive technologies, thus permitting a calculation of those choices in a trade-off between clinical intervention and efficiency and costs.

The results suggest the targeted use of POC testing technologies in communities and areas where prevalence might be higher than national average: e.g. MSM in Sydney, Melbourne and Brisbane can be done at extremely low cost with acceptable levels of effectiveness. Yet Australia has been slower than other countries at approving rapid testing technologies, in particular HIVST, despite an increased interest in testing using these devices [28–31]. HIVST is clearly less sensitive than either EIA or other rapid POC devices such as the DHC applied in this study as an initial screening tool, and will require, as with all regimes and settings, confirmation of an initial reactive result by the conventional pathway to diagnosis. Even so, HIVST is comparatively very cost effective as a part of delivering an eventual positive diagnosis. Cost savings are further available due to the low numbers of clients who will need confirmatory testing. If the price of HIVST drops due to ease of availability or volume production, then the savings it presents as a means of testing are potentially large.

### Limitations

This study found that the testing with rapid POC technologies are the most cost effective. However, there are two caveats to the results. First, the provability of having confirmation tests after Rapid POC testing regimes should be low; thus, the test efficiency could be lower and more costs might be required for the confirmation tests. Second, the number of false negative cases whilst not large would be sources of HIV transmission and might cause other costs.

## Conclusions

The trade-off between test effectiveness and cost-effectiveness may not necessarily be an area of charged medical ethical concern if POC testing is properly linked to pathways by which definitive diagnosis are given. This study advocates that a full suite of testing pathways including new and established testing technologies, especially POC, be employed in a manner that enables choices around testing and dispels barriers to testing for those at risk, and that new testing technologies should be deployed in an overall testing landscape that is cost-effective and sensitive to client preferences for testing. As such, the relative cost of setting and technology deployed, though of clear importance to the health system, should not solely determine public health strategies geared to increasing HIV diagnosis.

## Supplementary Information

**Additional file 1: Table A1.** Cost of Conventional Testing Regime: Private General Practice Clinic Setting – (Funded by Federal Government – Medicare). **Table A2.** Cost of Conventional Testing Regime: Public Sexual Health Clinic Setting (Funded by State Government). **Table A3.** Cost of Parallel Testing Regime: Community Peer Organisation Bulk Billing GP Clinic Setting (Funded by Federal Government - Medicare and State Government). **Table A4.** Cost of Parallel Testing Regime: Community Peer Testing Service using Volunteer Peer Nurse Setting (Funded by Federal Government - Medicare and State Government). **Table A5.** Cost of Point of Care Testing Regime: Community Peer Testing Service Setting (Funded by State Government). **Table A6a.** Cost of Point of Care Testing Regime: Home HIVST Setting (Includes Private Costs) (Funded by Consumer). **Table A6b.** Cost of Point of Care Testing Regime: Home HIVST Setting (Excludes Private Costs).

## Data Availability

The datasets used and/or analysed during the current study are available from the corresponding author on reasonable request.

## Abbreviations

ART: Antiretroviral treatment
DHC: Alere Determine™ HIV-1/2 Ag/Ab Combo Test
EIA: Enzyme-linked immunosorbent assay
HIV: Human Immunodeficiency Virus
HIVST: HIV self-testing
GP: General Practice
MSM: Men who have sex with men
PLHIV: People living with HIV
POC: Point of care
SHC: Sexual Health Clinic
STI: Sexually transmissible infections
WB: Western Blot
WoP: Whole of population
