# Supplementary material for: Economic evaluation of alternative testing regimes and settings to detect undiagnosed HIV in Australia
Source: BMC Health Serv Res. 2021 Jan 7;21:30. doi: 10.1186/s12913-020-06040-5 (PMC7789789; doi:10.1186/s12913-020-06040-5)
Supplement: Supplementary file 1 — Additional file 1: Table A1. Cost of Conventional Testing Regime: Private General Practice Clinic Setting – (Funded by Federal Government – Medicare). Table A2. Cost of Conventional Testing Regime: Public Sexual Health Clinic Setting (Funded by State Government). Table A3. Cost of Parallel Testing Regime: Community Peer Organisation Bulk Billing GP Clinic Setting (Funded by Federal Government - Medicare and State Government). Table A4. Cost of Parallel Testing Regime: Community Peer Testing Service using Volunteer Peer Nurse Setting (Funded by Federal Government - Medicare and State Government). Table A5. Cost of Point of Care Testing Regime: Community Peer Testing Service Setting (Funded by State Government). Table A6a. Cost of Point of Care Testing Regime: Home HIVST Setting (Includes Private Costs) (Funded by Consumer). Table A6b. Cost of Point of Care Testing Regime: Home HIVST Setting (Excludes Private Costs). [file 12913_2020_6040_MOESM1_ESM.docx]

Table A1 Cost of Conventional Testing Regime: Private General Practice Clinic Setting – (Funded by Federal Government – Medicare)

| **Whole of Population** | | | | |
| --- | --- | --- | --- | --- |
|  | **True HIV+** | | **True HIV-** | |
| **Outcome** | **HIV Positive (EIA+ & WB+)** | **False HIV Negative (EIA-)** | **False HIV Positive  (EIA+ & WB-)** | **True HIV Negative  (EIA-)** |
| Average Cost by Outcome | $ 221.90 | $ 159.05 | $ 188.05 | $ 159.05 |
| Probability of Outcome (%) | 0.0178 | 0.0000 | 0.1200 | 99.8622 |
| Average Cost by Outcome x Probability of Result | $ 0.0396 | $ 0.0000 | $ 0.2256 | $ 158.8308 |
| **Average Total Cost per Test by Regime and Setting** | $159.10 | | | |
| **Average Number of Tests for a Positive Diagnosis** | 5602.4656 | | | |
| **Cost per Positive Diagnosis**=Average Total Cost per Test * Average number of tests for a positive diagnosis | $891,329 | | | |

| **MSM** | | | | |
| --- | --- | --- | --- | --- |
|  | **True HIV+** | | **True HIV-** | |
| **Outcome** | **HIV Positive (EIA+ & WB+)** | **False HIV Negative (EIA-)** | **False HIV Positive  (EIA+ & WB-)** | **True HIV Negative  (EIA-)** |
| Average Cost by Outcome | $ 221.90 | $ 159.05 | $ 188.05 | $ 159.05 |
| Probability of Outcome (%) | 0.6040 | 0.0004 | 0.1193 | 99.2763 |
| Average Cost by Outcome x Probability of Result | $ 1.3404 | $ 0.0006 | $ 0.2243 | $ 157.8990 |
| **Average Total Cost per Test by Regime and Setting** | $159.46 | | | |
| **Average Number of Tests for a Positive Diagnosis** | 165.5527 | | | |
| **Cost per Positive Diagnosis**=Average Total Cost per Test * Average number of tests for a positive diagnosis | $26,399 | | | |

Table A2 Cost of Conventional Testing Regime: Public Sexual Health Clinic Setting (Funded by State Government)

| **Whole of Population** | | | | |
| --- | --- | --- | --- | --- |
|  | **True HIV+** | | **True HIV-** | |
| **Outcome** | **HIV Positive (EIA+ & WB+)** | **False HIV Negative (EIA-)** | **False HIV Positive  (EIA+ & WB-)** | **True HIV Negative  (EIA-)** |
| Average Cost by Outcome | $ 203.64 | $ 63.67 | $ 148.21 | $ 63.67 |
| Probability of Outcome (%) | 0.000178 | 0.000000 | 0.001200 | 0.998622 |
| Average Cost by Outcome x Probability of Result | $ 0.0363 | $ 0.0000 | $ 0.1778 | $ 63.5811 |
| **Average Total Cost per Test by Regime and Setting** | $63.80 | | | |
| **Average Number of Tests for a Positive Diagnosis** | 5602.4656 | | | |
| **Cost per Positive Diagnosis**=Average Total Cost per Test * Average number of tests for a positive diagnosis | $357,411 | | | |

| **MSM** | | | | |
| --- | --- | --- | --- | --- |
|  | **True HIV+** | | **True HIV-** | |
| **Outcome** | **HIV Positive (EIA+ & WB+)** | **False HIV Negative (EIA-)** | **False HIV Positive  (EIA+ & WB-)** | **True HIV Negative  (EIA-)** |
| Average Cost by Outcome | $ 203.64 | $ 63.67 | $ 148.21 | $ 63.67 |
| Probability of Outcome (%) | 0.00604 | 0.00000 | 0.00119 | 0.99276 |
| Average Cost by Outcome x Probability of Result | $ 1.2301 | $ 0.0002 | $ 0.1768 | $ 63.2081 |
| **Average Total Cost per Test by Regime and Setting** | $64.62 | | | |
| **Average Number of Tests for a Positive Diagnosis** | 165.5526 | | | |
| **Cost per Positive Diagnosis**=Average Total Cost per Test * Average number of tests for a positive diagnosis | $10,697 | | | |

Table A3 Cost of Parallel Testing Regime: Community Peer Organisation Bulk Billing GP Clinic Setting (Funded by Federal Government - Medicare and State Government)

| **Whole of Population** | | | | | | | | |
| --- | --- | --- | --- | --- | --- | --- | --- | --- |
|  | **True HIV+** | | | | **True HIV-** | | | |
| **Outcome** | **DHC+, EIA+, WB+** | **DHC-, EIA+, WB+** | **DHC+, EIA-** | **DHC-, EIA-** | **DHC+, EIA-** | **DHC+, EIA+, WB-** | **DHC-, EIA+, WB-** | **DHC- , EIA-** |
| Average Cost by Outcome | $ 232.90 | $ 170.05 | $ 232.90 | $ 98.35 | $ 170.05 | $ 199.05 | $ 199.05 | $ 98.35 |
| Probability of Outcome (%) | 0.0002 | 0.0000 | 0.0000 | 0.0000 | 0.0060 | 0.0000 | 0.0012 | 0.9926 |
| Average Cost by Outcome x Probability of Result | $ 0.04 | $ 0.00 | $ 0.00 | $ 0.00 | $ 1.02 | $ 0.00 | $ 0.24 | $ 97.63 |
| **Average Total Cost per Test by Regime and Setting** | $98.92 | | | | | | | |
| **Average Number of Tests for a Positive Diagnosis** | 5787.6713 | | | | | | | |
| **Cost per Positive Diagnosis**=Average Total Cost per Test * Average number of tests for a positive diagnosis | $572,542 | | | | | | | |

| **MSM** | | | | | | | | |
| --- | --- | --- | --- | --- | --- | --- | --- | --- |
|  | **True HIV+** | | | | **True HIV-** | | | |
| **Outcome** | **DHC+, EIA+, WB+** | **DHC-, EIA+, WB+** | **DHC+, EIA-** | **DHC-, EIA-** | **DHC+, EIA-** | **DHC+, EIA+, WB-** | **DHC-, EIA+, WB-** | **DHC- , EIA-** |
| Average Cost by Outcome | $ 232.90 | $ 170.05 | $ 232.90 | $ 98.35 | $ 170.05 | $ 199.05 | $ 199.05 | $ 98.35 |
| Probability of Outcome (%) | 0.0058 | 0.0000 | 0.0002 | 0.0000 | 0.0060 | 0.0000 | 0.0012 | 0.9868 |
| Average Cost by Outcome x Probability of Result | $ 1.36 | $ 0.00 | $ 0.05 | $ 0.00 | $ 1.01 | $ 0.00 | $ 0.24 | $ 97.05 |
| **Average Total Cost per Test by Regime and Setting** | $99.71 | | | | | | | |
| **Average Number of Tests for a Positive Diagnosis** | 171.0315 | | | | | | | |
| **Cost per Positive Diagnosis**=Average Total Cost per Test * Average number of tests for a positive diagnosis | $17,053 | | | | | | | |

Table A4 Cost of Parallel Testing Regime: Community Peer Testing Service using Volunteer Peer Nurse Setting (Funded by Federal Government - Medicare and State Government)

| **Whole of Population** | | | | | | | | |
| --- | --- | --- | --- | --- | --- | --- | --- | --- |
|  | **True HIV+** | | | | **True HIV-** | | | |
| **Outcome** | **DHC+, EIA+, WB+** | **DHC-, EIA+, WB+** | **DHC+, EIA-** | **DHC-, EIA-** | **DHC+, EIA-** | **DHC+, EIA+, WB-** | **DHC-, EIA+, WB-** | **DHC- , EIA-** |
| Average Cost by Outcome | $ 161.20 | $ 98.35 | $ 161.20 | $ 26.65 | $ 98.35 | $ 127.35 | $ 127.35 | $ 26.65 |
| Probability of Outcome (%) | 0.0002 | 0.0000 | 0.0000 | 0.0000 | 0.0060 | 0.0000 | 0.0012 | 0.9926 |
| Average Cost by Outcome x Probability of Result | $ 0.03 | $ 0.00 | $ 0.00 | $ 0.00 | $ 0.59 | $ 0.00 | $ 0.15 | $ 26.45 |
| **Average Total Cost per Test by Regime and Setting** | $27.22 | | | | | | | |
| **Average Number of Tests for a Positive Diagnosis** | 5,787.6713 | | | | | | | |
| **Cost per Positive Diagnosis**=Average Total Cost per Test * Average number of tests for a positive diagnosis | $157,566 | | | | | | | |

| **MSM** | | | | | | | | |
| --- | --- | --- | --- | --- | --- | --- | --- | --- |
|  | **True HIV+** | | | | **True HIV-** | | | |
| **Outcome** | **DHC+, EIA+, WB+** | **DHC-, EIA+, WB+** | **DHC+, EIA-** | **DHC-, EIA-** | **DHC+, EIA-** | **DHC+, EIA+, WB-** | **DHC-, EIA+, WB-** | **DHC- , EIA-** |
| Average Cost by Outcome | $ 161.20 | $ 98.35 | $ 161.20 | $ 26.65 | $ 98.35 | $ 127.35 | $ 55.65 | $ 26.65 |
| Probability of Outcome (%) | 0.0058 | 0.0000 | 0.0002 | 0.0000 | 0.0060 | 0.0000 | 0.0012 | 0.9868 |
| Average Cost by Outcome x Probability of Result | $ 0.94 | $ 0.00 | $ 0.03 | $ 0.00 | $ 0.59 | $ 0.00 | $ 0.15 | $ 26.30 |
| **Average Total Cost per Test by Regime and Setting** | $28.01 | | | | | | | |
| **Average Number of Tests for a Positive Diagnosis** | 171.0315 | | | | | | | |
| **Cost per Positive Diagnosis**=Average Total Cost per Test * Average number of tests for a positive diagnosis | $4,790 | | | | | | | |

Table A5 Cost of Point of Care Testing Regime: Community Peer Testing Service Setting (Funded by State Government)

| **Whole of Population** | | | | | | |
| --- | --- | --- | --- | --- | --- | --- |
|  | **True HIV+** | | | **True HIV-** | | |
| **Outcome** | **DHC+, EIA+, WB+** | **DHC-** | **DHC+, EIA-** | **DHC+, EIA+, WB-** | **DHC+, EIA -** | **DHC-** |
| Average Cost by Outcome | $ 277.90 | $ 33.50 | $ 215.05 | $ 244.05 | $ 215.05 | $ 33.50 |
| Probability of Outcome (%) | 0.0002 | 0.0000 | 0.0000 | 0.0000 | 0.0060 | 0.9938 |
| Average Cost by Outcome x Probability of Result | $ 0.05 | $ 0.00 | $ 0.00 | $ 0.00 | $ 1.29 | $ 33.29 |
| **Average Total Cost per Test by Regime and Setting** | $34.63 | | | | | |
| **Average Number of Tests for a Positive Diagnosis** | 5,787.6711 | | | | | |
| **Cost per Positive Diagnosis**=Average Total Cost per Test * Average number of tests for a positive diagnosis | $200,436 | | | | | |

| **MSM** | | | | | | |
| --- | --- | --- | --- | --- | --- | --- |
|  | **True HIV+** | | | **True HIV-** | | |
| **Outcome** | **DHC+, EIA+, WB+** | **DHC-** | **DHC+, EIA-** | **DHC+, EIA +,**  **WB-** | **DHC+, EIA-** | **DHC-** |
| Average Cost by Outcome | $ 277.90 | $ 33.50 | $ 215.05 | $ 244.05 | $ 215.05 | $ 33.50 |
| Probability of Outcome (%) | 0.0058 | 0.0002 | 0.0000 | 0.0000 | 0.0060 | 0.9880 |
| Average Cost by Outcome x Probability of Result | 1.6249 | 0.0065 | 0.0008 | 0.0017 | 1.2810 | 33.0977 |
| **Average Total Cost per Test by Regime and Setting** | $36.01 | | | | | |
| **Average Number of Tests for a Positive Diagnosis** | 171.0255 | | | | | |
| **Cost per Positive Diagnosis**=Average Total Cost per Test * Average number of tests for a positive diagnosis | $6,159 | | | | | |

Table A6a Cost of Point of Care Testing Regime: Home HIVST Setting (Includes Private Costs) (Funded by Consumer)

| **Whole of Population** | | | | | | |
| --- | --- | --- | --- | --- | --- | --- |
|  | **True HIV+** | | | **True HIV-** | | |
| **Outcome** | **HIVST+, EIA+, WB+** | **HIVST -** | **HIVST+, EIA-** | **HIVST+, EIA+, WB-** | **HIVST+, EIA -** | **HIVST-** |
| Average Cost by Outcome | $ 284.15 | $ 62.25 | $ 221.30 | $ 250.30 | $ 221.30 | $ 62.25 |
| Probability of Outcome (%) | 0.000164 | 0.000015 | 0.000000 | 0.000000 | 0.000200 | 0.999621 |
| Average Cost by Outcome x Probability of Result | $ 0.05 | $ 0.00 | $ 0.00 | $ 0.00 | $ 0.04 | $ 62.23 |
| **Average Total Cost per Test by Regime and Setting** | $62.32 | | | | | |
| **Average Number of Tests for a Positive Diagnosis** | 6,111.5584 | | | | | |
| **Cost per Positive Diagnosis**=Average Total Cost per Test * Average number of tests for a positive diagnosis | $380,860 | | | | | |

| **MSM** | | | | | | |
| --- | --- | --- | --- | --- | --- | --- |
|  | **True HIV+** | | | **True HIV-** | | |
| **Outcome** | **HIVST+, EIA+, WB+** | **HIVST -** | **HIVST+, EIA-** | **HIVST+, EIA+, WB-** | **HIVST+, EIA -** | **HIVST-** |
| Average Cost by Outcome | $ 284.15 | $ 62.25 | $ 221.30 | $ 250.30 | $ 221.30 | $ 62.25 |
| Probability of Outcome (%) | 0.005537 | 0.000503 | 0.000003 | 0.000000 | 0.000199 | 0.993757 |
| Average Cost by Outcome x Probability of Result | 1.5734 | 0.0313 | 0.0007 | 0.0001 | 0.0439 | 61.8614 |
| **Average Total Cost per Test by Regime and Setting** | $63.51 | | | | | |
| **Average Number of Tests for a Positive Diagnosis** | 180.5963 | | | | | |
| **Cost per Positive Diagnosis**=Average Total Cost per Test * Average number of tests for a positive diagnosis | $11,469 | | | | | |

Table A6b Cost of Point of Care Testing Regime: Home HIVST Setting (Excludes Private Costs)

| **Whole of Population** | | | | | | |
| --- | --- | --- | --- | --- | --- | --- |
|  | **True HIV+** | | | **True HIV-** | | |
| **Outcome** | **HIVST+, EIA+, WB+** | **HIVST -** | **HIVST+, EIA-** | **HIVST+, EIA+, WB-** | **HIVST+, EIA -** | **HIVST-** |
| Average Cost by Outcome | $ 221.90 | $ - | $ 159.05 | $ 188.05 | $ 159.05 | $ - |
| Probability of Outcome (%) | 0.000164 | 0.000015 | 0.000000 | 0.000000 | 0.000200 | 0.999621 |
| Average Cost by Outcome x Probability of Result | $ 0.04 | $ - | $ 0.00 | $ 0.00 | $ 0.03 | $ - |
| **Average Total Cost per Test by Regime and Setting** | $0.07 | | | | | |
| **Average Number of Tests for a Positive Diagnosis** | 6,111.5584 | | | | | |
| **Cost per Positive Diagnosis**=Average Total Cost per Test * Average number of tests for a positive diagnosis | $416 | | | | | |

| **MSM** | | | | | | |
| --- | --- | --- | --- | --- | --- | --- |
|  | **True HIV+** | | | **True HIV-** | | |
| **Outcome** | **HIVST+, EIA+, WB+** | **HIVST -** | **HIVST+, EIA-** | **HIVST+, EIA+, WB-** | **HIVST+, EIA -** | **HIVST-** |
| Average Cost by Outcome | $ 221.90 | $ - | $ 159.05 | $ 188.05 | $ 159.05 | $ - |
| Probability of Outcome (%) | 0.005537 | 0.000503 | 0.000003 | 0.000000 | 0.000199 | 0.993757 |
| Average Cost by Outcome x Probability of Result | 1.2287 | - | 0.0005 | 0.0000 | 0.0316 | - |
| **Average Total Cost per Test by Regime and Setting** | $1.26 | | | | | |
| **Average Number of Tests for a Positive Diagnosis** | 180.5963 | | | | | |
| **Cost per Positive Diagnosis**=Average Total Cost per Test * Average number of tests for a positive diagnosis | $227 | | | | | |
